# When Sepsis Mimics a Channelopathy: Brugada Phenocopy in a Patient With Urinary Tract Infection

**DOI:** 10.7759/cureus.107754

**Published:** 2026-04-26

**Authors:** Mehdi Chagdali, Najoua Mouina, Mouad Zakini, Zakia Touati, Mohamed Cherti

**Affiliations:** 1 Department of Cardiology B, Ibn Sina University Hospital, Rabat, MAR

**Keywords:** acquired brugada syndrome, brugada phenocopy, brugada syndrome, electrocardiogram, sepsis, urinary tract infection

## Abstract

Brugada phenocopies are clinical entities characterized by electrocardiographic patterns identical to those of congenital Brugada syndrome but occurring in the context of identifiable, reversible underlying conditions. Distinguishing between these two conditions is crucial, as their management and prognostic implications differ substantially. We report the case of a 71-year-old man with no prior cardiovascular history or family history of sudden cardiac death, who was admitted for confusion associated with general deterioration. The admission electrocardiogram (ECG) revealed a type 2 Brugada pattern. Laboratory findings demonstrated a significant inflammatory response, and an infectious workup confirmed a *Klebsiella pneumoniae* urinary tract infection. Serial ECGs showed complete normalization of the Brugada pattern following antibiotic therapy, consistent with a diagnosis of Brugada phenocopy. This case highlights the importance of considering Brugada phenocopy in patients presenting with Brugada-type ECG changes in the setting of acute infection, thereby avoiding unnecessary invasive interventions such as implantable cardioverter-defibrillator placement.

## Introduction

Brugada syndrome is a congenital cardiac channelopathy, characterized by persistent ST-segment elevation in the right precordial leads, and carries a significant risk of ventricular tachyarrhythmias and sudden cardiac death [1]. In contrast, Brugada phenocopies refer to electrocardiographic patterns that closely resemble those seen in true Brugada syndrome but arise from identifiable and reversible underlying conditions [2]. First formally characterized by Bayés de Luna et al. in 2012, these patterns share the same morphological classification, namely type 1 and type 2, as congenital Brugada syndrome [3]. However, they differ fundamentally in terms of their underlying causes and clinical implications.

Interest in Brugada phenocopies has grown steadily, though many questions remain unanswered. It is still unclear whether these patterns carry any intrinsic arrhythmic risk or predispose affected individuals to sudden cardiac death. As a result, there is no consensus on how best to manage patients who present with them [2]. What is clear, however, is the importance of distinguishing phenocopies from true Brugada syndrome, for which both prognostic expectations and therapeutic strategies are well established. Mistaking a phenocopy for true Brugada syndrome may lead to unnecessary interventions, such as the implantation of a cardioverter-defibrillator. Conversely, failing to recognize genuine Brugada syndrome could mean missing a critical opportunity to prevent life-threatening arrhythmic events [4].

It is important to note that not all Brugada-like ECG patterns are diagnostic. In particular, non-type 1 patterns (such as type 2) are not sufficient to establish a definitive diagnosis and require careful interpretation in the appropriate clinical context [2]. Furthermore, fever is a well-recognized trigger that may unmask latent Brugada syndrome, adding complexity to the diagnostic process [5]. Brugada phenocopies can be triggered by a wide range of conditions, including metabolic disturbances, myocardial ischemia, and pulmonary embolism. Infectious and inflammatory states have been less frequently reported, making such presentations relatively uncommon in clinical practice [6,7].

In this context, we report a case of a transient type 2 Brugada-like ECG pattern occurring in the setting of sepsis, which resolved after appropriate treatment, suggesting a Brugada phenocopy. This case highlights the diagnostic challenges associated with non-type 1 Brugada patterns in the presence of a reversible condition and underscores the importance of distinguishing between Brugada syndrome and Brugada phenocopy in order to guide appropriate management.

## Case presentation

A 71-year-old married man, father of five children, with no known modifiable cardiovascular risk factors, no significant past medical history, and no family history of sudden cardiac death, was admitted for the management of confusion that had developed two days prior, in the context of general deterioration, chills, and undocumented fever. Infectious history revealed dysuria, with no report of chest pain, palpitations, syncope, or presyncope.

On admission, the patient's Glasgow Coma Scale (GCS) score was 14/15. Vital signs were as follows: temperature 36.8°C, blood pressure 100/60 mmHg, heart rate 98 bpm, and oxygen saturation 95% on room air. The remainder of the physical examination was unremarkable.

**Figure 1:**
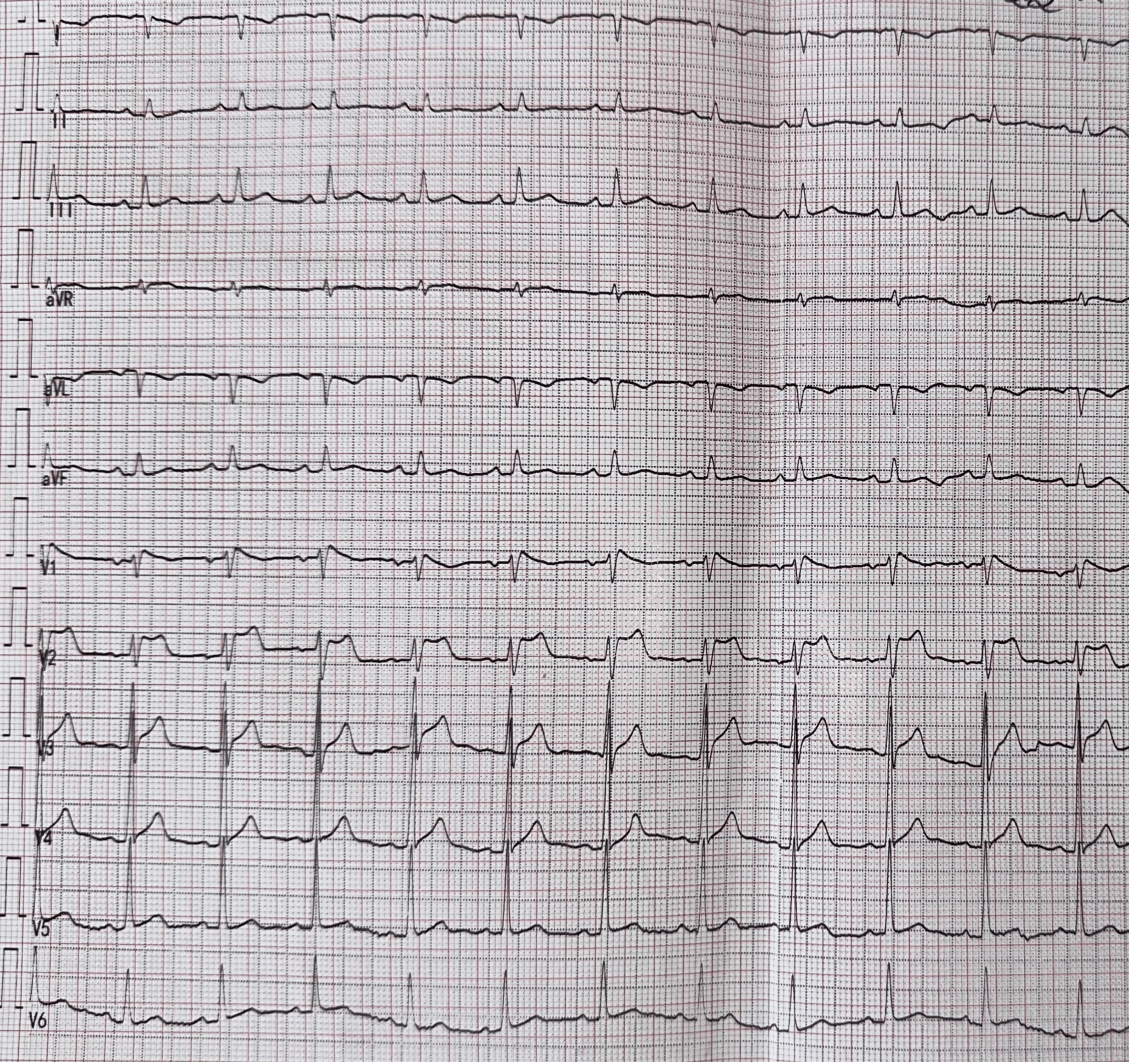
ECG on admission showing a type 2 Brugada pattern

The electrocardiogram (ECG) showed a regular sinus rhythm at 95 bpm, normal axis, and a right bundle branch block pattern in leads V1-V2, with an R' wave peak >2 mm above the isoelectric line. The descending limb of the R' wave exhibited a low slope (≥35°) with an ascending connection to the ST segment, resulting in a concave ST-segment elevation and a saddleback pattern (trough ≥0.5 mm above the isoelectric line) more pronounced in lead V2, followed by a negative T wave in V1 and a positive T wave in V2. The QRS complex was wider in V1 than in V6. These findings were consistent with a type 2 Brugada ECG pattern (Figure 1).

In order to increase diagnostic sensitivity and assess for a potential type 1 Brugada pattern, additional ECG recordings were performed with right precordial leads V1 and V2 positioned in higher intercostal spaces (second and third intercostal spaces). However, these recordings did not reveal any significant differences compared to the standard ECG, and no conversion to a type 1 Brugada pattern was observed.

Transthoracic echocardiography was unremarkable, revealing preserved global and segmental systolic function of the left ventricle, normal right ventricular function, and no pericardial effusion. Laboratory findings revealed a significant biological inflammatory syndrome suggestive of an infectious origin. Electrolyte levels and markers of myocardial necrosis were within normal limits (Table 1). Infectious workup revealed positive leukocyturia and bacteriuria on urine cytobacteriological examination, as well as a positive culture for *Klebsiella pneumoniae*.

**Table 1:** Biological test results of our patient on admission and at discharge CRP, C-reactive protein; HS, high sensitivity

Test	Results at admission	Results at discharge	Reference range
Total white cell (x10^3^/μL)	36	5.5	4–10
CRP (mg/L)	370.4	18	<5
Procalcitonin (ng/mL)	3.55	0.14	<0.05
Sodium (mEq/L)	143	139	136–145
Potassium (mEq/L)	4	4.6	3.5–5.1
Urea (g/L)	2.29	0.27	0.15–0.55
Creatinine (mg/L)	14.7	6.1	5.7–12.5
Troponin HS (ng/mL)	0.02	0.015	0.028–0.039

Given this clinical and biological presentation, several differential diagnoses were considered, including Brugada syndrome, Brugada phenocopy, acute coronary syndrome (ACS), and myocarditis. Further diagnostic workup with coronary angiography or cardiac magnetic resonance imaging was discussed. However, in the absence of chest pain, negative troponin levels on admission, and no ECG evidence of mirror-image changes or necrosis-related sequelae, the diagnoses of ACS and myocarditis were ruled out. Consequently, Brugada phenocopy and Brugada syndrome remained the primary diagnostic considerations.

**Figure 2:**
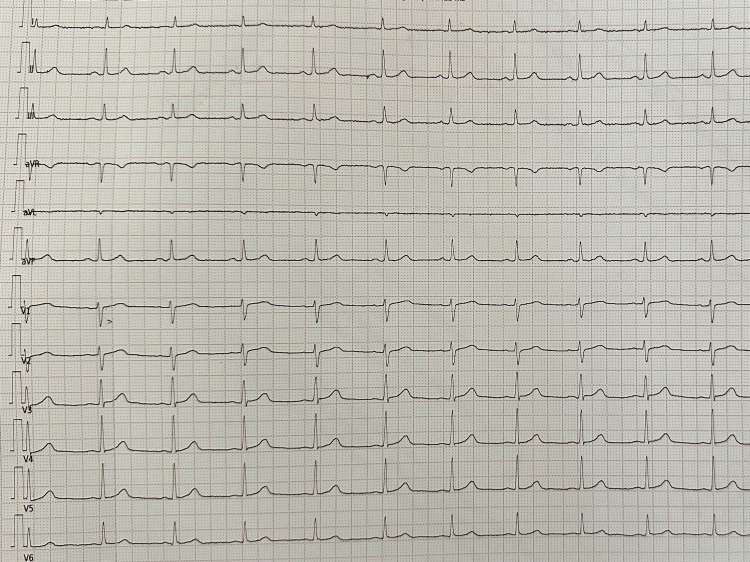
ECG showing resolution of the Brugada pattern after antibiotic treatment

Appropriate antibiotic therapy was initiated and maintained for 15 days, leading to favorable clinical and biological improvement (Table 1). The patient’s body temperature remained within normal limits from admission through discharge. Daily ECGs were performed, and the Brugada-like pattern resolved completely by the sixth day of antibiotic therapy (Figure 2). This ECG normalization occurred in parallel with clinical recovery and a marked decrease in inflammatory markers. Overall, this evolution was suggestive of a Brugada phenocopy related to the underlying septic condition rather than fever-induced unmasking of latent Brugada syndrome.

## Discussion

Brugada syndrome is a rare inherited cardiac disorder first identified approximately three decades ago. It is marked by distinct electrocardiographic abnormalities, including a pattern resembling right bundle branch block and persistent ST-segment elevation in the right precordial leads [8]. Three distinct repolarization patterns were initially identified. The type 1 ECG pattern is defined by a coved ST‑segment elevation of at least 2 mm followed by a negative T wave, with minimal or no isoelectric segment, and this morphology must be present in at least one right precordial lead (V1-V3). The type 2 pattern also shows ST‑segment elevation but is followed by a positive or biphasic T wave, giving a saddle‑back appearance. The type 3 pattern is characterized by a right precordial ST‑segment elevation of no more than 1 mm, which may exhibit either a coved or a saddle‑back morphology [9]. A definitive diagnosis of Brugada syndrome is made when, in addition to the typical ECG findings, the patient meets at least one of the following three criteria: a family history of sudden cardiac death in a relative younger than 45 years or a type 1 ECG pattern in a family member; a personal history of arrhythmia-related symptoms such as syncope, seizures, or nocturnal agonal respiration; or documented ventricular arrhythmias, including polymorphic ventricular tachycardia or ventricular fibrillation [10]. This condition is associated with an increased susceptibility to ventricular fibrillation and a significantly elevated risk of sudden cardiac death. Current estimates suggest that Brugada syndrome accounts for around 12% of all cases of sudden cardiac death and up to 20% of such deaths in individuals with no apparent structural heart abnormalities [5]. While some affected individuals remain entirely asymptomatic, the characteristic electrocardiographic pattern may appear either spontaneously or be unmasked by sodium channel blockers. Symptomatic patients, particularly those presenting with unexplained syncope, agonal breathing during sleep, or seizure-like episodes of unknown origin, face a heightened risk of sudden death and typically require implantation of a cardioverter-defibrillator [5].

Nevertheless, Brugada-like ECG patterns can occur in the absence of any underlying congenital ion channel dysfunction. This has led to considerable confusion regarding the appropriate terminology, with various authors using terms such as "acquired Brugada syndrome," "Brugada syndrome mimicry," and "Brugada-like ST segment abnormalities," among others. The term "Brugada phenocopy" was introduced by Riera et al. [11], and the condition was subsequently further characterized by Bayés de Luna et al. [3].

Brugada phenocopies are thought to arise from transient, reversible disturbances in cardiac electrophysiology. Proposed mechanisms include an imbalance between inward and outward ionic currents during phase 1 of the action potential, or delayed conduction in the right ventricular outflow tract. Increased transient outward potassium current or reduced sodium and calcium currents may create a transmural voltage gradient, particularly in the epicardium, promoting ST-segment elevation and phase 2 reentry. Unlike congenital Brugada syndrome, these changes are induced by reversible clinical conditions affecting ion channel function [12]. Among the reported triggers, metabolic imbalances, myocardial ischemia, and pulmonary embolism are the most commonly implicated, whereas sepsis has been described less frequently [7,13,14].

According to the International Registry, the diagnosis of Brugada phenocopy relies on a set of standardized criteria emphasizing electrocardiographic features and their clinical context, with cases categorized based on ECG morphology and the number of fulfilled criteria. A structured four-step approach has been proposed for suspected cases: first, identification of a Brugada-type ECG pattern; second, assessment of a low pretest probability for true Brugada syndrome based on clinical history; third, performance of a pharmacological challenge using a sodium channel blocker (such as ajmaline, flecainide, procainamide, or pilsicainide) to evaluate ECG response; and, finally, consideration of genetic testing, although this remains optional [15,16]. In our case, the first two criteria were fulfilled; however, the pharmacological challenge and genetic testing could not be performed due to the unavailability of sodium channel blockers and genetic testing facilities at our institution. Notably, the complete resolution of the Brugada pattern following treatment of the underlying infection supported the diagnosis of a Brugada phenocopy (Table 2).

**Table 2:** Summary table comparing Brugada syndrome and Brugada phenocopy Table Credits: Mehdi Chagdali. Data were derived from previously published studies [2,4,5,7,17]

Feature	Brugada Syndrome	Brugada Phenocopy
Nature of condition	Congenital cardiac channelopathy of genetic origin	Acquired entity mimicking the ECG pattern of Brugada syndrome induced by external factors
Etiology	Autosomal dominant mutation (principally in the *SCN5A* gene in 20-30% of cases)	Identifiable and reversible underlying conditions (e.g., hyperkalemia, hyponatremia, acidosis, ischemia, mechanical compression, or sepsis)
ECG profile	Persistent or dynamic repolarization abnormalities (type 1, 2, or 3)	Transient Brugada-like pattern on the surface ECG
Clinical History	Often associated with a family history of sudden cardiac death or syncope	Low pre-test probability; absence of family history of sudden cardiac death or unexplained syncope
Provocative Testing	Positive (unmasks a type 1 pattern after administration of a sodium channel blocker)	Negative (a mandatory diagnostic criterion to exclude latent Brugada syndrome)
Resolution	The pattern may fluctuate, but the underlying genetic predisposition is permanent	Spontaneous resolution of the ECG pattern once the underlying metabolic or structural cause is corrected
Standard treatment	Implantation of an implantable cardioverter-defibrillator for high-risk patients	Direct treatment of the underlying cause

## Conclusions

Distinguishing Brugada syndrome from Brugada phenocopies is of paramount clinical importance, as the two entities carry fundamentally different prognostic implications and therapeutic strategies. While Brugada syndrome may require implantable cardioverter-defibrillator placement due to arrhythmic risk, phenocopies are reversible upon resolution of the underlying cause. Misdiagnosis can lead to unnecessary invasive procedures or missed preventive opportunities. A systematic approach integrating clinical context, electrocardiographic evolution, and standardized diagnostic criteria is crucial. This case highlights the importance of recognizing phenocopies to guide appropriate management and avoid the potential harms of misclassification.
